# Protein family comparison using statistical models and predicted structural information

**DOI:** 10.1186/1471-2105-5-183

**Published:** 2004-11-25

**Authors:** Richard Chung, Golan Yona

**Affiliations:** 1 Department of Computer Science, Cornell University, Ithaca, NY 14850, USA

## Abstract

**Background:**

This paper presents a simple method to increase the sensitivity of protein family comparisons by incorporating secondary structure (SS) information. We build upon the effective information theory approach towards profile-profile comparison described in [Yona & Levitt 2002]. Our method augments profile columns using PSIPRED secondary structure predictions and assesses statistical similarity using information theoretical principles.

**Results:**

Our tests show that this tool detects more similarities between protein families of distant homology than the previous primary sequence-based method. A very significant improvement in performance is observed when the real secondary structure is used.

**Conclusions:**

Integration of primary and secondary structure information can substantially improve detection of relationships between remotely related protein families.

## Background

Detecting an evolutionary relationship between proteins is the basis for functional inference. Existing methods most often rely on sequence information in an attempt to quantify the evolutionary divergence or similarity between the sequences compared. A significant similarity would suggest that the proteins are related. However, in many cases sequences have diverged to the extent that their similarity is undetectable by standard sequence comparison algorithms. Nevertheless, they may still have similar structures and functions [1,2].

It has long been postulated that evolutionary pressure acts upon the three-dimensional structure of proteins and intra-protein interactions rather than at the level of the primary sequence [3,4]. Indeed, there is plenty of evidence to suggest that 3D structure is more conserved than sequence [5,6]. Since the protein structure usually prescribes the function of a protein, relying on structural information (for example, through structure comparison) for functional inference is more effective and reliable than using only the primary sequence. However, although methods of sequencing proteins have become faster and more cost-efficient due to recent technological advancements, methods to determine structure are still in their infancy. In fact, less than 5% of newly sequenced proteins have a known structure. Current empirical processes used to determine structure of proteins are neither efficient nor scalable to use upon the entire known protein space.

There have been many attempts to build algorithms that predict protein structure from amino acid sequence. Unfortunately, this is a hard problem, and existing methods are only partially successful [7]. On the other hand, predicting the secondary structure of a protein has been more successful. There are various algorithms that predict the secondary structure from primary amino acid sequence information alone [8-13]. The accuracies of these algorithms have been steadily increasing, and one of the most successful algorithms to date is PSIPRED [13], which has an average accuracy of about 80%. Since the architecture of the secondary structure elements of a protein affects its global structure, it has been suggested that secondary structure information can be used to detect subtle similarities between proteins that have diverged substantially in the course of evolution. This principle was tested in [14] where a dynamic programming algorithm with a secondary-structure based scoring matrix was used to compare protein sequences over the alphabet of secondary structures. However, relying solely on secondary structure information might lead to poor performance overall, as much of the original information about the individual amino acids is lost.

Alternatively, one can use both representations to assess protein similarity. Incorporating secondary structure information into protein comparison is not a new idea. Several researchers have attempted to boost performance and sensitivity of various methods by adding this extra degree of information with some success. Yu et al. encoded functionally conserved sequence patterns into probabilistic structural models (that comprise a family of hidden Markov models) [15]. The models were benchmarked against the trypsin-like serine protease family and the globin family, and in both cases proved to have high specificity and sensitivity compared to the models in use at the time (primarily, BLAST) in remote homology detection. One of the limitations of this model, however, was the reliance on threading methods requiring at least one determined structure to build a model. Hedman et al. [16] included information about predicted transmembrane segments into the standard Smith-Waterman and profile-search algorithms for membrane proteins by adding an extra delta (score) when two residues that are both predicted to belong to transmembrane segments are aligned. This method was found to improve the detection rate, mainly by increasing specificity (ie. decreasing the number of false positives). Ginalski et al. [17] generalized a method of creating "meta profiles" by combining sequence information with predicted secondary structure information. Total scores were calculated by summing the raw score obtained from the shifted dot product of the sequence profile vectors and the shifted dot product of the secondary structure probability vectors (weighted by some factor). This technique was derived from hybrid threading approaches and was found to be more sensitive than the sequence-only approach or sequence-to-structure threading approach. Teodorescu et al. [18] proposed a linear combination of threading and sequence-alignment to produce a single (mixed) scoring table. This method was found to be particularly sensitive in detecting sequences with less than 25% of sequence identity, yet with similar structures. The final model outperforms the individual scoring methods.

These and similar studies have indicated that the incorporation of secondary structure information, even if predicted, can increase sensitivity and specificity of a protein comparison model. Here we describe a method that integrates secondary structure information with primary sequence information in a single scoring scheme, using a single statistical representation. The model can be applied to any protein family and does not require the application of expensive threading algorithms. Our method extends our previous work on profile-profile comparison [19]. Specifically, we use the profile representation (generated by PSI-BLAST) as a statistical model of a protein family and augment the profile with structural information. We then compare profiles of different protein families, in search of possible remote kinship, using an information theory-based scoring function. By comparing models of protein families we can detect similarities that are not detected when comparing individual sequences. We show that the new algorithm improves performance and can detect more similarities between remote protein families. These similarities can be used to classify protein families into super-families and detect higher order structure within the protein space.

## Methods and Results

### Data sets

We use a data set of domain families derived from the SCOP classification of protein structures [20], release 1.50. This set contains 23,780 protein domains classified into 1,287 protein families. Each of the 1,287 families is represented by a profile that was generated using PSI-BLAST [21]. The seed of the profile was selected to be the sequence whose average distance from all other members of the family is the smallest. Families for which there is only one member, or for which PSI-BLAST failed to generate a profile, were represented by a profile generated directly from the seed sequence by using probabilities derived from the original BLOSUM62 frequency matrix [22]. A subset of 456 families was used in our study, all of which belong to superfamilies that contain at least 3 families. A smaller subset of 120 families was used for parameter optimization.

### Sequence profiles

The PSI-BLAST profiles are the basis for our representation of a protein family. Each profile is a *n*-tuple of probability distributions of amino acids, derived from a group of related proteins, where *n *is the length of the multiple alignment of these proteins. It is represented in software as a two dimensional matrix of 20 rows and *n *columns, where each column (known as a profile column), is a probability distribution **p **over the 20 amino acids in one position in the multiple alignment. These profile columns form the basis of profile-profile comparisons.

### Secondary structure information

We use two types of secondary structure information in our experiments: true information and predicted information. The *true *secondary structure information is gleaned from the PDB files of the seed proteins using STRIDE [23]. Stride defines eight types of secondary structures *b*, *B*, *C*, *E*, *H*, *I*, *G*, *T *where b and B stand for Bridge, C = Coil, E = Strand, H = AlphaHelix, I = PiHelix, G = 310Helix and T = Turn. We reduce this set to the three main secondary structures (helix, strand and coil) by mapping H, I, G to H, and b, B, C, T to C.

The *predicted *secondary structure information is predicted using PSIPRED [13]. PSIPRED uses the intermediate sequence profiles generated by PSI-BLAST as input for the prediction algorithm. This profile matrix is fed into a standard feed-forward back-propagation neural network with a single hidden layer using a window of 15 residues. This net has three output units corresponding to each of the three states of secondary structures. Another window of 15 positions of these three outputs (per amino acid) are then used as input into a second neural network to filter and smooth outputs from the main network. The final output is the probability that a certain amino acid position in the seed sequence of a profile is in a coil, helix, or strand. PSIPRED reports an average Q3 score of approximately 80% accuracy.

### Integrating secondary structure with primary structure

Apriori, it is unclear how one should integrate secondary structure with primary structure in a single model. For example, one might think of a representation over a generalized alphabet, that considers all possible pair combinations of amino acids and secondary structure elements. Assuming independence between positions (which is the underlying assumption of position specific scoring matrices, as well as of HMMs that are used in computational biology), then this representation implies that for each position *i *we have a statistical source that emits amino acid *a *and secondary structure *s *with probability *P*_*i*_(*a*, *s*) such that


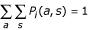


and every position can be represented by a vector of 60 probabilities over this pair alphabet. This representation implicitly implies that the amino acid emitted and the secondary structure are two different features of the objects generated by the source, while in reality the secondary structure is not a "character" or an independent property of the emitted objects, but rather a characteristic of the source itself that is usually unknown. This property introduces some special constraints on the distribution of amino acids that are emitted by the source. In other words, the secondary structure and the amino acid distribution in a position are strongly dependent on each other, but one is *hidden *while the other is *visible*. Noting that *P*_*i*_(*a*, *s*) can be written as *P*_*i*_(*a*/*s*)*P*_*i*_(*s*), we can decompose the parameter space into the parameters of the secondary structure distribution, and the parameters of the conditional probability distributions over amino acids. However, the typical amino acid distributions that are available from multiple alignments of protein families differ from these conditional probability distributions by definition. Furthermore, there are other subtleties that one should bear in mind when designing an integrated statistical model for a protein family.

More precisely, assume we have a protein family, where all proteins adopt a certain structural conformation of length *n*. This conformation can be described in terms of the set of 3D coordinates of the *n *positions, or in terms of the set of distances between coordinates **S **= (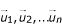) where 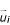 is the set of distances from the *i*-th residue to all other residues – the latter being more amenable for a representation as a statistical source, as it is invariant to translations and rotations. Although there is structural variation across the different instances of the protein family, it is significantly smaller than the sequence variation, and we will assume that a single *consensus *conformation **S **reliably describes the protein family. The structural conformation determines the statistical properties of the source distributions. Namely, it induces certain constraints on the sequence space that can be mapped to that conformation, based on the physical properties of its topology. In other words, it induces a probability distribution over the sequence space of *O*(20^*n*^) sequences that can be mapped to that conformation

*P*(*a*_1_, *a*_2_, ..., *a*_*n*_/**S**).

Note that due to convergent evolution it is possible that two disconnected regions in the sequence space (two families of homologous proteins) will be mapped to the same conformation (although experimental evidence and simulation results [24] suggest that this is not very likely, and for most protein families it is reasonable to assume that the sequence space that is mapped to a structural conformation is connected). This 20^*n*^-dimensional distribution clearly introduces dependencies between remote positions, and the exact probability distribution in a position depends on the amino acids observed in all other positions *P*(*a*_*i*_/*a*_1_, *a*_2_, ..., *a*_*i*-1_, *a*_*i *+ 1_, ..., *a*_*n*_, **S**). Accurate knowledge of the all-position probability distribution *P*(*a*_1_, *a*_2_, ..., *a*_*n*_/**S**) would allow one to compare two sources of protein families theoretically by comparing these high-dimensional distributions. However, because of (limited) data availability and for mathematical simplicity, the *marginal probabilities*


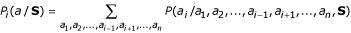


are usually used in practice to describe the source. Given a multiple alignment of a specific protein family, and the corresponding profile, the empirical distribution of amino acids at position *i*, denoted by 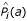, is essentially the marginal probability of amino acids at that position, as *constrained by the global conformation*, i.e.


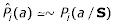


The complete model is represented as a set of marginal probability distributions, one per position. So far we have not considered the secondary structures explicitly. The secondary structure sequence s is a reduced representation of **S **that, while incomplete, describes quite accurately the topology of the protein. Given **S**, the knowledge of s however does not affect the distribution of amino acids at a position, i.e.

*P*_*i*_(*a*/**s**, **S**) = *P*_*i*_(*a*/**S**)

Nevertheless, the secondary structure information can still be useful when comparing protein families. This is because some information is lost if one is to use just the *marginal *amino acid distributions. For example, the same marginal amino acid distribution can be observed in different secondary structure conformations and on the other hand, even highly similar fragments of secondary structures can be associated with different amino acid distributions.

The most effective way of comparing two protein families is by comparing their (consensus) structural conformations **S**_**1 **_and **S**_**2**_. Indeed, it has been shown that structure comparison is much more effective in detecting remotely related families [19,20,25]. In statistical terms, one can formulate the problem of comparing consensus structures **S**_**1 **_and **S**_**2 **_as comparing two sources that induce different probability distributions over the conformation space *P*^1^(**S**) and *P*^2^(**S**). However, characterizing these distributions is very difficult. Moreover, convergent evolution might result in two *different *sequence sources with structurally similar conformations. These relations are usually perceived weaker than families that are similar both in sequence and structure [20]. Therefore, a proper comparison should account for both the primary and tertiary structure. In statistical terms, we are interested in comparing the joint distributions 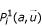 and 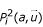, where the distributions are again marginalized over all positions other than *i*, and 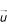 is a vector of inter-residue distances. The joint distributions can be rewritten as


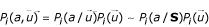


where the last step uses the more accurate marginal probabilities *P*_*i*_(*a*/**S**) that are based on all vectors of inter-residue distances (and match the empirical distributions 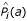).

As was mentioned earlier, obtaining **S **is difficult (and therefore also characterizing the distributions of inter-residue distances). On the other hand, secondary structure (which can be viewed as an approximation of **S**) is more readily available, and can be predicted quite reliably from sequence information. Therefore we suggest to approximate


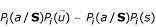


where *P*_*i*_(*s*) is the probability to observe a secondary structure s at the *i*-th position. (When the secondary structure is known the distribution over secondary structures assigns probability 1 to one of the structures and zero otherwise. However, with predicted information, each state is usually assigned a non-zero probability based on the amino acids in that position and neighboring positions.) Plugging in the empirical distributions 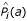 for *P*_*i*_(*a*/**S**) we get


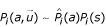


i.e., the empirical distribution of amino acids at a position, 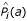, is conditionally independent of the distribution *P*_*i*_(*s*). Therefore, to completely describe the source one needs to provide the parameters of the marginal distribution of amino acids, *and *the parameters of the secondary structure distribution. Since the two distributions are assumed independent, they are amenable to a representation in which their parameters are appended together. I.e. the secondary structure probabilities are appended to the probabilities of the 20 amino acids.

Our method is based on an extension of the original profile representation in [19]. Using the three PSIPRED probabilities, we augment the profile columns of primary information to make a probability distribution over 23 values (the 20 amino acids plus 3 secondary structures). Note that by doing so, each profile column is now dependent upon and contains information about its neighbors, since PSIPRED uses the profile columns surrounding each profile column to deduce the probability that the position in question is in a specific secondary structural conformation. This is the key element that enhances the accuracy of this tool in protein family comparisons. Moreover, the method is "self-contained" in the sense that for the secondary structure prediction, PSIPRED uses the same profiles that are generated by PSI-BLAST. To use the profile-profile metrics described next, the 23-dimensional profile columns have to be normalized to conform with probability distributions. However, apriori it is not clear if the primary information and the secondary structure information should be weighted equally. To control the impact of the secondary structure information on the representation we introduce a mixing parameter *γ *that ranges from 0 to 1. The secondary structure probabilities are normalized such that they sum to *γ *while the amino acid probabilities are normalized such that they sum to 1 - *γ*. The higher *γ *is, the more dependent the profile column is upon secondary structure information. This parameter is optimized as described in section 'Parameter optimization'.

Note that our normalization maintains the conditional independence of the two types (primary and secondary), as described above. Each component of the extended profile can be viewed as a sub-profile. Since each one of the two components is summed independently to a non-zero probability then two symbols must be "emitted": an amino acid *and *a secondary structure.

### Profile-Profile comparison

In this section we review the main elements of our profile-profile comparison algorithm that was introduced in [19]. We compare profiles using the dynamic programming algorithm with an information theoretic-based scoring function to score pairs of profile columns. Given two profiles **P **= **p**_**1**_**p**_**2**_**p**_**3**_**...p**_**n **_and **Q **= **q**_**1**_**q**_**2**_**q**_**3**_**...q**_**m**_, where *n *and *m *are the lengths of the profiles (the number of positions or columns) and **p**_**i**_, **q**_**j **_are probability distributions over the 23 letter alphabet of amino acids and secondary structures, we define the similarity score between two columns **p**_**i **_and **q**_**j **_based on their statistical similarity. The similarity score is composed of two elements: the divergence score and the significance score.

#### The divergence score

To estimate the divergence of two probability distributions we use the Jensen-Shannon (JS) divergence measure [26]. Given two (empirical) probability distributions **p **and **q**, for every 0 ≤ *λ *≤ 1, the *λ*-JS **divergence **is defined as


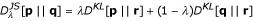


where *D*^*KL*^[**p**||**q**] is the Kullback-Leibler (KL) divergence [27], defined as


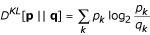


and

**r **= *λ***p **+ (1 - *λ*)**q**

can be considered as the most likely common source distribution of both distributions **p **and **q**, with *λ *as a prior weight (here set to 0.5). We call the corresponding measure the **divergence score **and denote it by *D*^*JS*^. This measure is symmetric and ranges between 0 and 1, where the divergence for identical distributions is 0. Besides being symmetric and bounded, an attractive feature of the *D*^*JS *^divergence measure is that it is proportional to the minus logarithm of the probability that the two empirical distributions represent samples drawn from the same ("common") source distribution [28]. It has also been shown that 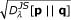 is a metric [29].

#### The significance score

The divergence score measures one aspect of the statistical similarity of **p **and **q**: their relative distance. However, it does not consider the uniqueness of the two distributions. A match between two distributions that resemble the background distribution is not as significant as a match of two distributions that resemble each other, but are very different from the background distribution. In other words, the more unique the distributions are (and hence, also their common source), the more significant is a match between them.

To assess the **significance score ***S *of a match we measure the JS divergence of the (common) source distribution, **r**, from the base (background) distribution *P*_0_.

*S *= *D*^*JS*^[**r**||**P**_**0**_]

In this study the background distribution is composed of two components: the background distribution of amino acids (estimated from a large sequence database) and the background distribution of secondary structure elements (estimated from all PDB structures). The components are mixed using the same mixing parameter *γ *described in section 'Integrating secondary structure with primary structure'. The significance measure reflects the probability that the source distribution, **r**, could have been obtained by chance. The higher **r **is, the more distinctive the common source distribution, and the lower the probability that it could have been obtained by chance.

#### The similarity score

We define the **similarity score **of two probability distributions **p **and **q **as a combination of the divergence score and the significance score:


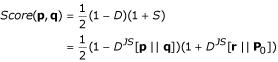


With this expression, the similarity score of two similar distributions (*D *→ 0) whose common source is far from the background distribution (*S *→ 1), tends to one. On the other hand, the similarity score of two dissimilar distributions (*D *→ 1) whose most likely common source distribution resembles the background distribution (*S *→ 0) tends to zero. This scoring scheme also distinguishes two distributions that each are similar to the background distribution (*D *→ 0 and *S *→ 0 giving *Score *- 1/2) from two dissimilar distributions, but whose common source is similar to the background distribution (*D *→ 1 and *S *→ 0 giving *Score *= 0). In a recent study [30] it has been shown that this scoring function is one of the best, when compared to other methods for profile-profile comparison.

Note that our measures are functionals of the probability distributions, based on variations of the entropy function, and specifically the KL divergence function. One of the nice properties of this function is that it is additive in the following sense. Assume we have a probability distribution **p **over a set *X *that is obtained by "mixing" two probability distributions over two disjoint subsets: **p**_**1 **_over the subset *X*1 and **p**_**2 **_over the subset *X*2 (where *X *= *X*1 ∪ *X*2 and *X*1 ∩ *X*2 = *θ*). Let *γ *be the mixing parameter, i.e. the total weight of the first distribution **p**_**1 **_in the combined distribution **p**. Assume **q **is obtained in a similar manner from **q**_**1 **_and **q**_**2**_. Then,


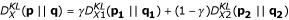


In other words, this measure preserves independence between the two subsets. Therefore, with our extended profile representation, the new functionals are simply a weighted sum of the individual functionals over the subsets *X*1 (the secondary structure) and *X*2 (the primary structure).

Note however that this property holds for the divergence and the significance measures but not for the final similarity score that is a combination of the divergence and the significance scores. An alternative would be to compute the divergence, significance and similarity scores independently for the secondary and primary structures, and then combine the two similarity scores into one, with weights *γ *and (1 - *γ*) respectively.

### The effect of secondary structure on the pairwise scores

It is interesting to compare the similarity scores before and after the addition of secondary structure information. To assess the impact of this information, we computed the distribution of similarity scores for five types of profile columns, depending on the type of their seed amino acid. We refer to the amino acid at position *i *of the seed sequence (see section 'Data sets') as the **seed amino acid **of the *i*-th profile column.

Two seed amino acids are defined as similar, neutral, or dissimilar based on their BLOSUM62 scoring matrix [22], with positive, zero and negative substitution scores respectively.

**Figure 1:**
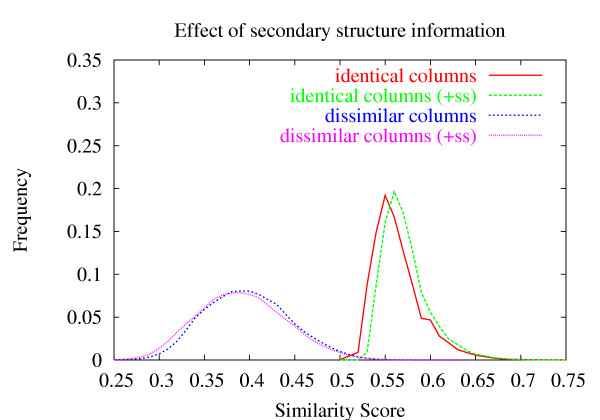
**Distribution of *similarity scores *before and after adding secondary structure information **for identical columns (a column with itself) and dissimilar columns.

The five types of column pairs are: (1) a column with itself (**identical columns**), (2) different columns that are associated with the same seed amino acid (**strongly similar columns**) (3) different columns that are associated with similar seed amino acids (**similar columns**), (4) different columns with mutually neutral seed amino acids (**neutral columns**), and (5) different columns with dissimilar seed amino acids (**dissimilar columns**). We repeated this calculation before and after the integration of true secondary structure information (using the optimal mixing parameter *γ*, see section 'Parameter optimization') and the results are plotted in Figure 1. As the figure indicates, there is a slight shift between the distributions, and the addition of secondary structure information pushes the distributions further apart, decreasing the distribution overlap, as desired. Although the differences are small (due to the very small value of the optimal mixing parameter), the effect on the performance is significant as is demonstrated in section 'Discussion'.

### Comparison of scoring functions

We compared our information-theoretic scores to other popular scoring schemes. We tested the **correlation scores **based on the scalar product of the vectors (as was suggested in [31]).


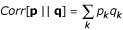


We also tested the **ALLR **(Average Log Likelihood Ratio) scoring function that was suggested in [32]. This scoring function is also based on information-theoretic principles, and resembles ours. Given two empirical probability distributions **p **and **q**, their ALLR score is defined as


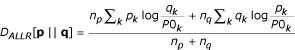


where *n*_*p *_(*n*_*q*_) is the number of total counts from which **p **(**q**) is derived, and **P0 **is the background distribution.

**Figure 2:**
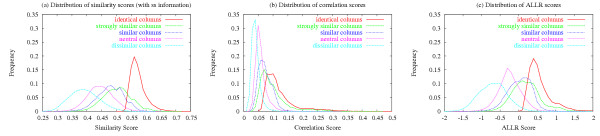
(a) **Distribution of *similarity scores *for different column types. **(b) **Distribution of *correlation scores***. (c) **Distribution of *ALLR scores. ***The distributions are based on the largest 100 families in SCOP 1.50 database. The pairs of profile columns are divided into five categories depending on the nature of the seed amino acids, as described in the text. Note that in general the distributions of *correlation scores *overlap more than the other scoring functions, and specifically, the overlap between the scores of identical columns and the scores of dissimilar columns is greater (24%) than the overlap between the same types of columns, using our similarity scores (2.1%) or the ALLR scores (4.4%).

We computed the correlation scores and ALLR scores for the same sets of columns defined in the previous section and compared it to the information-theoretic scores (Figure 2). Note that the correlation scores are less successful in distinguishing related columns from columns which are likely to be unrelated (compare Figure 2a and Figure 2b). The overlap is larger and the tail of the fifth distribution (dissimilar columns) falls well within the first distribution (identical columns). Specifically, 24% of the pairs of dissimilar columns have correlation scores that overlap with scores of identical columns, compared to only 2.1% when using our similarity scores. We believe that this may affect the performance significantly. On the other hand, the ALLR scores have very similar properties to ours, although the overlap between dissimilar columns and identical columns is greater (4.4%).

### Parameter optimization

Our algorithm (*prof*_*ss*) depends on several parameters: (1) a shift parameter is introduced to convert the similarity scores to scores that are suitable for local protein comparison (other transformations were tested in [19] and proven less effective); (2) gap penalties for the dynamic programming algorithm; (3) the mixing parameter *γ*

#### Shift parameter and gap penalties

as Figure 2a shows, the distributions of identical columns (red line) and distributions with dissimilar seed amino acids (black line) are quite well separated around 0.5. In addition, distributions with mutually neutral seed amino acids peak at a similarity score around 0.45. Note that the new similarity scores (after the addition of the secondary structure information) preserve the overall behavior (quantitatively and qualitatively) as the old similarity scores (see Figure 1). The mean of the scores is unchanged and only the variance has increased. Therefore, we decided to maintain the same set of parameters that were optimized in [19]. Specifically, we used the same shift value of 0.45 and the same gap penalties of 2 (gap opening) and 0.2 (gap extension). We have also tested position-specific gap penalties based on the SS information, but without any apparent improvement in performance.

#### Mixing parameter

**Figure 3:**
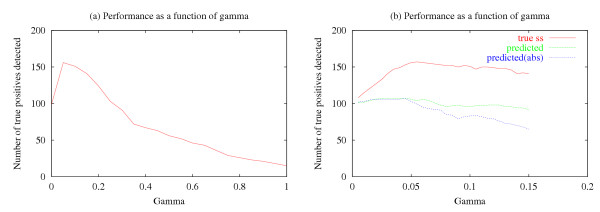
**The effect of the mixture parameter *γ *on the performance. **Performance is measured by the number of true relations that are detected before the first false positive (see section for more details), **(a) **Exploring *γ *in the range [0,1] using *prof*_*ss *with true secondary structure information, **(b) **Magnification of the range 0–0.15. The peak is obtained at *γ *= 0.055. Approximately the same value is obtained when using *prof*_*ss *with PSIPRED predicted secondary structure information and also when using predicted information after the probabilities were rounded such that the most probable state is assigned the probability of 1 and the other two states are assigned probability of 0.

To estimate the best value for *γ *we used a subset of 120 families and assessed the performance for different values of *γ*. Our performance evaluation procedure works as follows: true relationships are defined to be between those families that share a superfamily and all others are defined as false relationships. For each family within the test set, we calculate the profile-profile similarity against all 1287 families for a single value of the mixing parameter *γ*. These results are sorted by raw score and the number of true family-family relationships are counted before the first false relationship is detected (this is basically a sum of ROC1 scores). The tradeoff between the primary sequence information and secondary structure probabilities was varied from zero to one. With zero dependence on secondary structure the method is equivalent to *prof*_*sim *(profile-profile comparison based on just primary structure). The results are shown in Figure 3. As the graph indicates, setting *γ *= 0.055 (i.e. 0.055 weight on the secondary structure information and 0.945 on the sequence information) gave the best performance. (Note that if each secondary structure was given as much weight as a single amino acid *γ *would be 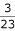 or ~0.13).

When only secondary structure information was used (*γ *= 1), the performance was much worse than when only sequence information was used (*γ *= 0). These corner-case results and the fact that the best results were obtained with *γ *<< 0.5 suggest that for protein family comparison, the coarse-grained secondary structure information is noisier and less reliable than sequence information. However, as the graphs indicate, using both sources of information clearly improves performance. Our tests were done using actual secondary structure information in the profile; however, similar results were obtained when the predicted information was used for one or both of the profiles (see Figure 3b).

### Statistical significance

To differentiate true similarity values from those that may be observed by chance, it is essential to establish a baseline empirical distribution for the scores. Here we used the statistical framework of the extreme value distribution (EVD). Although rigorous mathematical proof has not been found for local gapped similarity scores, empirical studies have shown that the distribution of these scores can be approximated by this distribution. An empirically fit EVD also has the benefits of being a true fit to the quirks of a particular protein family. Three such distributions were established to assess the significance of the profile-profile matches. All distributions were fit with the 'fit' function in gnuplot  using the nonlinear least-squares (NLLS) Marquardt-Levenberg algorithm.

**Figure 4:**
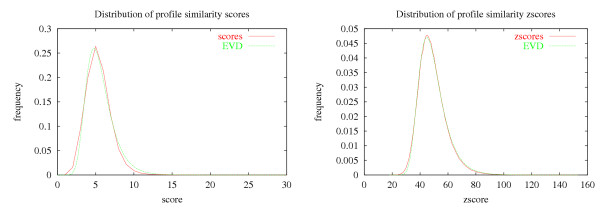
**Distribution of profile similarity scores. (a) **The uniform approach: the scores follow the EVD distribution (with *λ *= 0.707 and *u *= 4.77. **(b) **The normalized length-corrected scores (zscores) also follow the EVD (zscores are shifted and multiplied by 10 to emulate typical values for raw similarity scores).

The first distribution is based upon comparisons between unrelated families (defined as families that belong to different SCOP classes and do not share significant structural similarity). This distribution is useful in that it can be used to assess the significance of a score in comparing any pair of protein families, without further need for computations. Practically, this aggregates all comparisons between non-related families into a single list. This is essentially the distribution of similarity scores of random profiles, as shown in Figure 4a. By fitting an EVD to this distribution we can estimate the statistical significance (e-value) of any raw similarity score. We refer to this method as the *uniform approach *(uniform parameters).

The second distribution is similar to the first, except a correction was made for the length of a profile, similar to the approach employed by FASTA [33]. By chance, the raw score of a profile-profile comparison is greater for those profiles with many more residues than the score of two smaller profiles. To correct for this occurrence, all raw scores were fit to a logarithmic curve of the product of the two profile lengths. The mean and variance of this fit was used to calculate a zscore. Accounting for undersampling at the ends of the spectrum, the means were fit to a linear curve and the variance was constant throughout. The distribution of zscores was then fit to an EVD, as is shown in Figure 4b. This distribution estimates better the statistical significance of raw similarity scores since it accounts for the biases introduced due to the lengths of the profiles.

**Figure 5:**
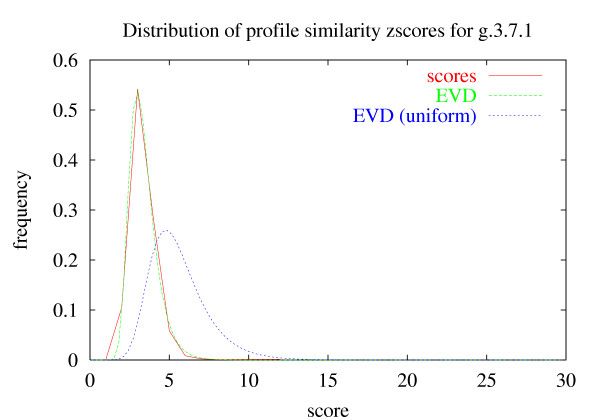
**Distribution of profile similarity scores. **The per-family distribution can differ quite markedly from the overall distribution of scores due to unique composition effects, etc, as is demonstrated for the g.3.7.1 family.

The third distribution proves to be the best approach in assessing significance of matches with a particular profile. This distribution is created on a per-family basis. The scores of each family against all (unrelated) SCOP families were fit to an EVD. Since many of the family profiles are unrelated to the query family, the corresponding scores provide a relatively reliable baseline distribution. This approach is a robust method to assessing the significance of matches for a particular profile since it allows for any unusual properties of the query profile (like unusual amino acid composition) and the parameters are adjusted accordingly (see Figure 5). Once again, from the fitted EVD, the e-value of the raw similarity scores is estimated from this fit.

**Figure 6:**
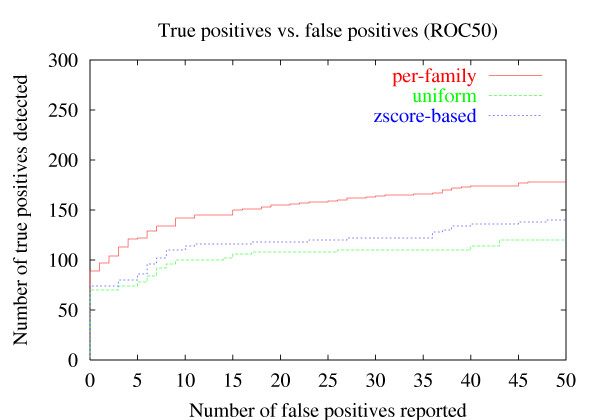
**Comparison of different methods for assessing pvalue. **For each method we plot the ROC50 curve (see section for details). Note that the zscore-based approach is better than the uniform approach, and is outperformed by the per-family approach.

The third method of measuring statistical significance is self-calibrating and provably more accurate than the previous two methods, and our performance evaluation tests indicate that this is the best method overall (see Figure 6). However, it is an intractable method when given a single pair of profiles to compare, since there is no prior knowledge about the baseline distributions of either profile. As a result, we must rely on the second method to measure statistical significance in these cases.

## Discussion

We evaluate the performance of our algorithm using the SCOP database as a benchmark and two measures of performance. The first counts the number of weak relationships between protein families (as implied by the SCOP classification) that can be detected with our method. Specifically, each family in our test set is compared with all other protein families and the results are sorted based on the p-value. Given the sorted list we count the number of true family-family relationships that are detected before the *first *false positive occurs. This measure is applied to each family *individually*, and the results, summed over all families in the test set are given in Table 1. We compare our results to Gapped-BLAST, PSI-BLAST and *prof*_*sim *(as reported in [19]).

Usually a false positive is defined as a relation between families that do not belong to the same superfamily. This popular criterion, however, is somewhat strict as relations between families that belong to the same fold can also be considered as positives. We use the following terminology to distinguish between the different types of "false positives". We define a relationship between two protein families to be a **true relationship **if both families belong to the same superfamily, a **possible relationship **if both families belong to the same SCOP fold, a **weak relationship **if they belong to the same class, **suspicious **if they belong to different classes (excluding the case of an all-alpha ↔ all-beta pair) and an **error **if one family is all-alpha and the second is all-beta. We repeat the procedure described above, each time using a different definition of a false positive. The results are summarized in Table 1.

The second measure we use is the receiver operator characteristic (ROC) measure, a common measure in assessing sensitivity and selectivity. Given a sorted list of results, the ROC index measures the area under the curve that plots the positives versus the negatives. Maximal performance translates to a perfect separation and a maximal normalized ROC score of 1. The ROC-N measure is a variation over the ROC measure, where the plot is truncated at N negatives. In other words, the ROC-N measure is the number of true positives detected up to N false positives. Here we used the popular ROC-50 measure. To obtain the ROC-50 scores for each method we pool together all pairwise comparisons for all protein families, and sort them by their normalized e-value. The number of true positives is aggregated until 50 false positives occur. As before, we repeated this procedure with different definitions of false positives, and the results are summarized in Table 2. A detailed break-up of the pairwise similarities detected with each method is given in Table 3 (using the most strict definition of a false positive). Note that *prof*_*ss *improves over *prof*_*sim *(for all types of false positives) although the improvement is smaller compared to the one reported in Table 1. The difference in performance is striking when the true secondary structure information is used. Despite the moderate contribution to the profile (the optimal *γ *was set to 0.055), the new algorithm almost doubles the number of pairwise relationships that are detected.

### Examples

In this section we give several interesting examples of alignments between remote protein families that exemplify the differences between sequence-based profile-profile alignments and the new generalized profile alignments.

#### The "winged helix" DNA-binding domain superfamily

**Figure 7:**
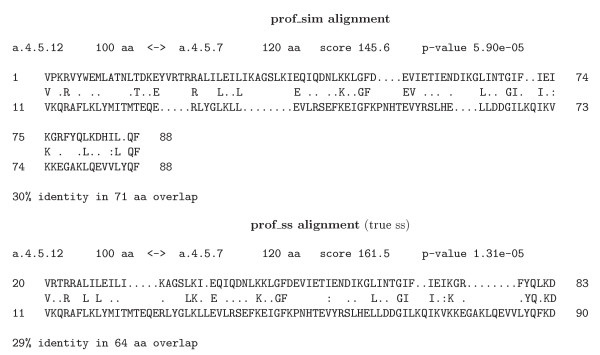
Alignment of SCOP families a.4.5.12 and a.4.5.7

**Figure 8:**
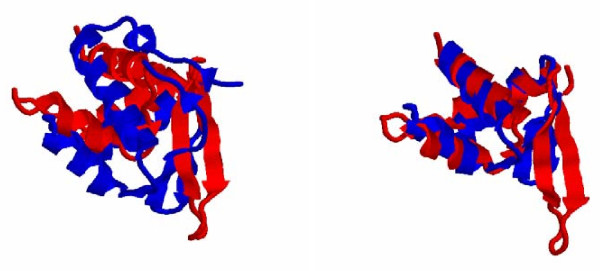
**Structural superposition of SCOP families a.4.5.12 and a.4.5.7 based on Profile-Profile alignment. (a) ***prof*_*sim *alignment (rms 11.96) **(b) ***prof*_*ss *alignment (rms of 4.45).

This superfamily is part of the DNA/RNA-binding 3-helical bundle fold. We compared two families from that superfamily: the restriction endonuclease FokI, N-terminal recognition domain (family a.4.5.12, seed scop domain d2foka3), and the replication terminator protein (family a.4.5.7, seed scop domain dlbm9a_). Although designated as all-alpha, proteins in this superfamily contain a small beta-sheet at the core. The similar substructures have three alpha helices and a couple beta strands, *prof*_*sim *is able to roughly match up the helices but not the beta strands with a rms of 11.96. The predicted secondary structure does not improve the alignment in this case, however, when the true secondary structure is used, *prof*_*ss *is able to completely align the helices as well as most of the strands with a much better rms of 4.45 (Figure 7 and Figure 8).

#### The concanavalin A-like lectins/glucanases superfamily

This superfamily belongs to the concanavalin A-like lectins/glucanases fold, characterized by a sandwich structure with 12–14 strands in 2 sheets. We compared two families in this superfamily: the beta-Glucanase-like family (b.29.1.2, seed domain dlcpm__) and the vibrio cholerae sialidase, N-terminal and insertion domains (b.29.1.8, seed domain dlkit_2).

**Figure 9:**
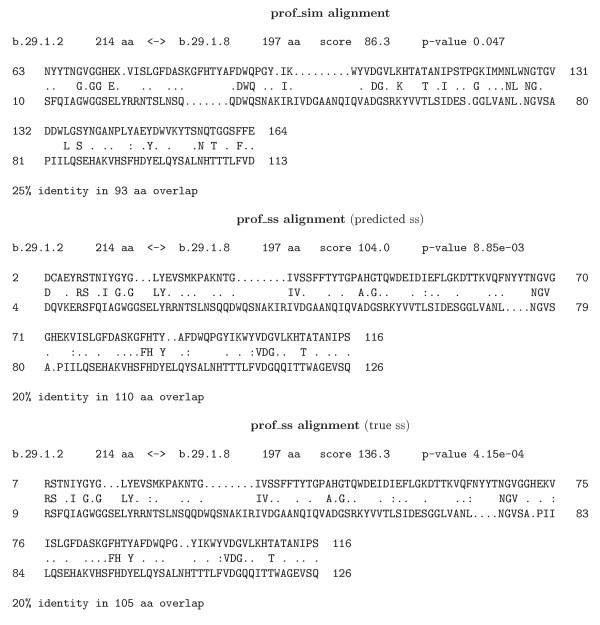
Alignment of SCOP families b.29.1.2 and b.29.1.8

**Figure 10:**
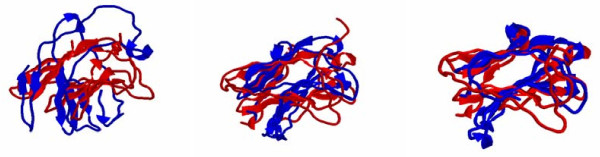
**Structural superposition of SCOP families b.29.1.2 and b.29.1.8 based on Profile-Profile alignment**. **(a) ***prof*_*sim *alignment **(b) ***prof*_*ss *alignment (predicted ss). **(c) ***prof*_*ss *alignment (true ss).

These class beta proteins have complex topology and are hard to align even with structure alignment algorithms. In this example, the two sets of beta sheets are nicely aligned by *prof*_*ss *both when using the predicted information and the true secondary structure information. On the other hand, *prof*_*sim *is unable to align the sheets at all (see Figure 9 and Figure 10).

#### The alpha/beta-Hydrolases superfamily

**Figure 11:**
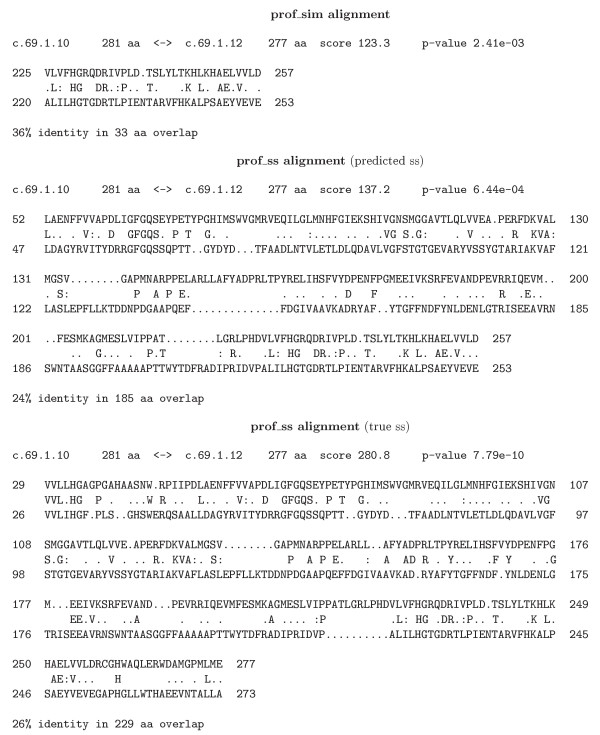
Alignment of SCOP families c.69.1.10 and c.69.1.12

**Figure 12:**
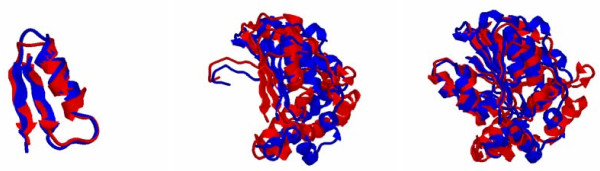
**Structural superposition of SCOP families c.69.1.10 and c.69.1.12 based on Profile-Profile alignment. (a) ***prof*_*sim *alignment **(b) ***prof*_*ss *alignment (predicted ss). **(c) ***prof*_*ss *alignment (true ss).

The alpha/beta-Hydrolases belong to the fold by the same name. Proteins with that fold are composed of 3 layers at the core, of alpha/beta/alpha. We compared two families in this superfamily: the carbon-carbon bond hydrolase family (c.69.1.10, seed domain dlc4xa_) and the bromoperoxidase A2 family (c.69.1.12, seed domain dlbrt__). These are large and complex proteins with many helices and strands. *prof*_*sim *reports an alignment that aligns perfectly one small alpha helix and two beta strands. With predicted secondary structures, *prof*_*ss *is able to generate a much longer alignment, with *γ *alpha helices and 4 beta strands. The alignment is not perfectly in sync, but all secondary structures are roughly in position. When using the true secondary structure information in *prof*_*ss *the alignment improves and a better overlap is observed (see Figure 11 and Figure 12).

## Conclusion

This paper presents a simple method to improve remote homology detection between protein families. We use statistical models of protein families in the form of profiles, and by incorporating secondary structure information within that model, we can reuse existing comparison methods for comparing profiles. It is shown that this method improves over the previous method that is based only on primary sequence information.

As opposed to other methods that compare single proteins, our method compares models of protein families. Instead of summing over different models, our model combines structural and primary sequence information within the profile itself. Our method allows us to explore a wide range of scenarios, between purely sequence-based representation and a purely secondary-structure based representation. The optimization of the single mixing parameter shows that the slight incorporation of predicted secondary structural information is invaluable. Since predicted structure information in PSIPRED comes from neighboring profile columns, this proves that each profile column confers extra information that is relevant to its neighbors and is useful to inferring protein relationships.

Furthermore, it is shown that if true secondary structure information is used, performance improvements are very significant and the number of relationships that can be detected is almost doubled. We conclude that despite the high overall accuracy of the secondary structure prediction method, its imperfect nature can greatly affect the performance. However, our method can be generalized to any secondary structure prediction method that produces estimated probabilities for secondary structure, so should a new prediction method be found that performs better than the current methods, the model presented here is expected to reflect the improved performance and consequently improve homology detection.

## Authors' contributions

RC extended the *prof*_*sim *program and integrated secondary structure information, optimized the model, ran experiments, and analyzed the result sets. GY conceived of the study, designed the model and analyzed the results.

## Figures and Tables

**Table 1:** Performance evaluation results (ROC1). Methods compared: Gapped BLAST, PSI-BLAST, *prof*_*sim *and *prof*_*ss*. For each method, the number of true family-family relationships that are detected before the first false connection occurs, is given (applied individually to each family, and summed over all families in the test set). Results are given for the following types of false connections: possible, suspicious and error (see text for details).

	Number of true	family-family	relationships	detected by	
Type of first false-relation	Gapped-BLAST	PSI-BLAST	*prof*_*sim*	*prof*_*ss *(predicted ss)	*prof*_*ss *(true ss)

Different superfamily, same fold ("possible" relationship)	163	189	231	245	391
Different fold, same class ("weak" relationship)	163	191	231	245	398
Different class ("suspicious" relationship)	174	205	253	299	483
Alpha ↔ Beta ("error" relationship)	709	690	1586	1617	1771

**Table 2:** Performance evaluation results (ROC50). Our test set is composed of 456 families that have at least 2 related families within the same superfamily (see section 'Data sets'). For each method, we report the number of *true *relationships that are detected until 50 false connections occur. We repeat this analysis four times, each time with a different definition of a false positive. A break up of the relationships by relationship-type is given in Table 3 for the first type of false positive.

	Number of true	family-family	relationships	detected by	
Type of false-positive	Gapped-BLAST	PSI-BLAST	*prof*_*sim*	*prof*_*ss *(predicted ss)	*prof*_*ss *(true ss)

Different superfamily, same fold ("possible" relationship)	116	146	173	178	272
Different fold, same class ("weak" relationship)	116	148	173	178	274
Different class ("suspicious" relationship)	124	153	189	209	328
Alpha ↔ Beta ("error" relationship)	214	268	308	>395	>584

**Table 3:** Performance evaluation results (break up of ROC50 results). For each method, we report the number of true, possible, suspicious and error relationships that are detected until 50 false positives occur (here, a false positive is any relationship between families that do not belong to the same superfamily). Also given are the e-value at which the 50th error occurs. The last column lists the number of relationships that are detected with *prof*_*ss *when using the true secondary structure assignments.

	Number	of	relationships	detected by	
Relationship-type	Gapped-BLAST	PSI-BLAST	*prof*_*sim*	*prof*_*ss *(predicted ss)	*prof*_*ss *(true ss)

Same superfamily (true relationship)	116	146	173	178	272
Same fold ("possible" relationship)	0	2	1	1	6
Same class ("weak" relationship)	18	17	17	26	31
Different class ("suspicious" relationship)	31	30	31	23	13
Alpha ↔ Beta ("error" relationship)	1	1	1	0	0
Total	166	196	223	228	322
e-value	0.1	0.1	0.39	0.34	0.33
